# Untargeted metabolomics profiles delineate metabolic alterations in mouse plasma during lung carcinoma development using UPLC-QTOF/MS in MS^E^ mode

**DOI:** 10.1098/rsos.181143

**Published:** 2018-09-19

**Authors:** Huan Wu, Yang Chen, Zegeng Li, Xianhua Liu

**Affiliations:** 1 Key Laboratory of Xin'an Medicine, Ministry of Education, Anhui Province Key Laboratory of R&D of Chinese Medicine, Anhui University of Chinese Medicine, Hefei 230038, People's Republic of China; 2 Institute of Pharmaceutics, Anhui Academy of Chinese Medicine, Hefei 230012, People's Republic of China; 3 National Key Disciplines of Lung Disease of Anhui University of Chinese Medicine, State Administration of Traditional Chinese Medicine, Hefei 230038, People's Republic of China; 4 Department of Chinese Medicine, the First Affiliated Hospital of Anhui Medical University, Hefei 230022, People's Republic of China

**Keywords:** untargeted metabolomics, lung carcinoma, UPLC-QTOF-MS, multivariate data analysis, receiver operating characteristic curve

## Abstract

In this work, an untargeted metabolomic method based on ultra-high-performance liquid chromatography-quadrupole time-of-flight mass spectrometry (UPLC-QTOF/MS) in MS^E^ (E represents collision energy) mode was exploited to determine the dynamic metabolic alterations in the plasma of male C57BL/6 mice during the onset and development of lung carcinoma. Plasma samples were collected from control and model mice (male C57BL/6 mice experimentally inoculated with the *Lewis* lung carcinoma cells) at 7 and 14 days post-inoculation (DPI). As a result, 15 dysregulated metabolites, including cholesterol sulphate, tiglylcarnitine, 1-palmitoylglycerophosphoinositol, 2-stearoylglycerophosphoinositol, stearoylcarnitine, PC(20:2(11Z,14Z)/16:0), PC(22:4(7Z,10Z,13Z,16Z)/14:0), PC(22:5(7Z,10Z,13Z,16Z,19Z)/14:0), PC(22:6(4Z,7Z,10Z,13Z,16Z,19Z)/16:0), 12,20-Dioxo-leukotriene B4, sphingosine 1-phosphate(d19:1-P), sphingomyelin(d18:0/16:1(9Z)), lysoPC(16:0), lysoPC(18:0) and lysoPC(20:4(5Z,8Z,11Z,14Z)), were identified in the plasma of model mice with xenografts at both 7 and 14 DPI. All the altered metabolites associated with the onset and development of lung carcinoma were involved in the metabolism of glycerophospholipid, fatty acid, sphingolipid and arachidonic acid. The feasible utility of these endogenous biomarkers as potential diagnostic indicators was validated through receiver operating characteristic curve analysis. Collectively, these findings provide a systematic view of metabolic changes linked to the onset and development of lung carcinoma.

## 1. Introduction

Lung carcinoma is the most common cause of cancer mortality in China [1]. The global incidence was almost two million new cases in 2012 [2], and more than one-third of these new cases were diagnosed in China [3]. The 5-year survival rate of lung carcinoma in the early operable stage is approximately 66–82%. However, when the tumour spreads to distant sites, the 5-year survival rate drops to 6% [4]. Therefore, the early diagnosis and staging of lung carcinoma is crucial to determine treatment plans and guide disease prognosis [5]. Currently, diagnosis mainly depends on the clinical symptoms, and detection frequently occurs at an advanced stage, thus leading to a very poor prognosis. If diagnosis could be accomplished in earlier stages, the overall morbidity and mortality would dramatically change.

Recent studies have reported that low-dose computed tomography screening for lung carcinoma patients is effective to reduce the mortality of disease [6]. Nevertheless, there was a large number of individuals with undefined nodules and limitations in the resources available to identify more precise risk profiles, ideally based on minimally invasive or non-invasive means (i.e. exhaled air, phlegm, blood, etc.) [7], in conjunction with clinical symptoms, imaging, lifestyle and epidemiological information. The above diagnosis strategy could be especially applicable to individuals at risk of lung carcinoma as they may have clinical disease for years before their presentation.

Untargeted metabolomics, as an analytical tool used in combination with analytical platform and multivariate data analysis, has emerged as a reliable approach in systems biology [8]. Its objective being the comprehensive profiling of low-molecular-weight endogenous metabolites *in vivo*, mining the most significant metabolites that play crucial roles in biological pathways linked to disease pathogenesis and providing fundamental insights into the molecular mechanisms involved in the onset and development of diseases [9,10]. Currently, the most commonly employed analytical platforms used to profile metabolic alterations are mass spectrometry (MS) and nuclear magnetic resonance (NMR) spectroscopy. Although high-resolution NMR affords decisive structural information and quantitative analysis of metabolites *in vivo*, it suffers from deficiencies in chemical separation and sensitivity. In contrast, given their high accuracy, high sensitivity and specificity, and large dynamic range, high-resolution hybrids mass analysers such as Orbitrap and quadrupole time-of-flight (QTOF) MS are adequate to obtain more information in complex biological matrices [11].

Although some promising results have been studied in the metabolic alterations of lung carcinoma [12–14], there is still a lack of a systematic understanding of biological information during the onset and development of lung carcinoma *in vivo*, such as a wide range of metabolic levels, which is pivotal to illustrate the pathology of the disease. Therefore, a comprehensive method for the detection of the onset and development of lung carcinoma requires broad metabolites in the more representative set of biological matrices that could provide a basic consideration for the discovery of biomarkers. Peripheral blood (plasma or serum), the most commonly used biological matrix in untargeted metabolomics, possesses a huge amount of metabolite information and has been shown to delineate the entire pathophysiological mapping for the early stages of some oncological diseases [15]. In this study, an untargeted metabolomic method based on ultra-high-performance liquid chromatography (UPLC)-QTOF in MS^E^ (E represents collision energy) mode was explored to globally profile and identify the specific and reliable endogenous biomarkers linked to the onset and development of lung carcinoma in male mouse plasma. Both multivariate data analysis and univariate statistics were used to detect the reproducibility of the analysis based on the detected ionic strength and by rapidly mining the interesting metabolites. Finally, modules of enrichment analysis and metabolic pathway analysis were employed to elucidate the possible biological mechanisms of the endogenous biomarkers associated with the onset and development of lung carcinoma.

## 2. Experimental procedures

### 2.1. Chemicals and reagents

Chromatographic-grade formic acid was purchased from SIGMA Chemical & Co (St Louis, USA). MS grade acetonitrile and methanol were purchased from MERCK & Co., Inc. (Darmstadt, Germany). Ultra-high purity water was produced through Millipore-Q purification system (Bedford, USA).

### 2.2. Tumour strain

The *Lewis* lung carcinoma tumour strains were purchased from the cell repository in the Biological Sciences Institute of Shanghai and then cultured in Dulbecco's modified Eagle's medium/high glucose (37°C) in a saturated humidity incubator containing 5% CO_2_ [16].

### 2.3. Animal model

All protocols and care of the mice were performed in strict compliance with Guidelines for the Use of Laboratory Animals (National Research Council) and authorized by the Animal Care and Use Committee of Anhui University of Chinese Medicine. Thirty male C57BL/6 mice (eight weeks old) were bought from the Animal Center of Anhui Medical University (Hefei, China). All C57BL/6 mice were acclimated at 55 ± 10% humidity and 20 ± 0.5°C on a reverse 12/12 h lamp switching cycle in an animal breeding room under specific pathogen-free (SPF) conditions. Sterilized chow and purified water were provided *ad libitum*. Mice were adapted for one week prior to the initial experiment. To establish xenograft tumours of *Lewis* lung carcinoma, *Lewis* lung carcinoma cells (2 × 10^6^) were inoculated into the right forelimb of each mouse by subcutaneous injection (S.C.) [17]. After the implantation, 20 model mice were randomized into two groups: a 7-day post-inoculation (DPI) model group and 14 DPI model group. Sarcoma growth was monitored every 7 days. The Vernier scale calliper was used to measure perpendicular diameters of the sarcoma. The sarcoma volume was calculated as follows: sarcoma size = long diameter × (short diameter)^2^/2.

### 2.4. Sample collection and preparation

All mice were fasted overnight before sample collection. Ten mice in each group were anaesthetized and sacrificed. Blood was collected through the retro-orbital area into heparin anticoagulation tubes. Then, the tubes were centrifuged at 2500*g* for 6 min at 4°C to obtain the plasma. The mice were executed, and sarcomas were dissected and weighed. Volumes of 100 µl plasma were aliquoted and transferred to 1.5 ml Eppendorf (Ep) tubes. At the same time, a quality control pooled (QCP) sample was prepared by mixing 10 µl of each test sample.

Small molecule metabolites were extracted from plasma fractions after adding methanol to remove macromolecules [18]. Frozen samples were thawed at 4°C for 0.5 h. Then, 100 µl aliquots of plasma were deproteinized with 400 µl cold methanol in a 1.5 ml Ep tube and vortex mixing for 60 s. The mixture was centrifuged at 13 000*g* for 5 min at 4°C. The supernatant was obtained and filtered via a microporous membrane (0.22 µm) and transferred into a sampling vial. A 2 μl aliquot of each vial was injected for the following UPLC-QTOF/MS analysis.

### 2.5. Chromatographic and mass spectrometric conditions

The analysis was performed on an ACQUITY I-Class UPLC equipment coupled with a Xevo G2-XS QTOF/MS detector (Waters Corp. Milford, MA, USA) via an electrospray interface. The chromatographic separation of all samples was performed on an ACQUITY UPLC BEH C_18_ column (Waters Corp.) (dimension 100 × 2.1 mm, 1.7 µm particle size). The temperature of column and auto-sampler were maintained at 48°C and 4°C, respectively. The UPLC system runs a gradient elution program consisting of water with 0.1% formic acid (solvent A) and acetonitrile (solvent B). The linear gradient was optimized and described as follows: 0 min, 8% B; 4 min, 40% B; 19 min, 85% B; 24–26 min, 95% B; 27–30 min, 8% B, which was delivered at 0.2 ml min^−1^.

Mass spectrometry analysis was conducted in positive and negative ion modes. The optimized conditions of QTOF/MS were: capillary voltage, 3.0 kV/−2.5 kV; source temperature, 120°C; sampling cone, 40 kV; cone gas flow, 50 l h^−1^; desolvation temperature and flow rate were 350°C and 600 l h^−1^; scan range, 50–1200 *m/z*; data acquisition rate, 0.5 s. MS^E^ model was selected for acquisition: the lower collision energy was 6 V, the higher collision ramp energy was 15–40 V. To ensure the accuracy, the *m/z* values of all ions acquired in the QTOF/MS were real-time adjusted by LockSpray. Leucine-enkephalin was selected as lock mass compound for positive ion mode ([M + H]^+^ = 556.2771) and negative ion mode ([M − H]^−^ = 554.2615). In order to balance the UPLC-MS system, the QCP sample was repeatedly injected three times before the formal sampling to ensure system equilibrium. And then it was injected again at the beginning, re-injected at every five samples and at the end of the sample collection (total of seven injections) to further monitor the reproducibility of the analytical platform.

### 2.6. Data processing and analysis

The raw data were acquired using Masslynx 4.1 Workstation UPLC-QTOF/MS Acquisition software (Waters Company, Milford, MA, USA) in non-targeted mode. After acquisition, QTOF/MS raw data were imported to Progenesis QI v. 3.0.3 software for automatic data processing [19]. The detailed workflow for data processing and analysis included retention time correction, experimental design set-up, peak picking, normalization, deconvolution, the identification of compounds and statistics. Retention time (*t*_R_)–*m/z* datasets were used to characterize the detected ions. The ‘30% rule in QCP samples’ and ‘80% rule in test samples’ were used to filter the consistent ions [20].

Then, the generated *t*_R_–*m/z* datasets and their corresponding ion intensities were further exported to EZinfo for multivariate data analysis. Principal component analysis (PCA) was employed to validate the technical reproducibility of the analytical approach and visualize the clustering, trends and outliers among the variables [21]. Orthogonal partial least squares discriminant analysis (OPLS-DA) was applied to screen differential variables responsible for the differentiation between the groups after Pareto scaling [22].

The loading-plots and S-plots generated from the OPLS-DA model were employed to visualize the relative importance of the differential variables and acquire a list of peak indices. Variables at VIP (variable influence on projection) with a value larger than 1.0 and fold change larger than 1.2 were selected and further input into *Welch's t*-test to test the meaning of each feature. The areas under the receiver operating characteristic curves (ROC) were employed to assess the significance of the biomarkers [23]. In general, only *t*_R_–*m/z* pairs with VIP > 1, fold change > 1.2 and *p* < 0.05 were considered as the potential biomarkers.

To identify the chemical structures of the metabolites, the accurate *m/z* was first derived to match the metabolite from the online Metlin [24], HMDB [25] and LipidBlast [26] databases. A potential biomarker was sieved out when the difference of the parent and fragment ions between the detected and calculated exact mass was no more than 5 ppm.

## 3. Results and discussion

### 3.1. Characteristics of *Lewis* lung carcinoma mice

**Figure 1.**
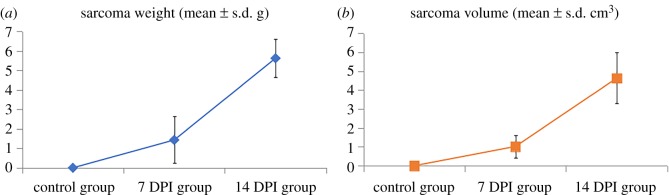
The sarcoma weights (*a*) and volumes (*b*) of the control, 7 DPI and 14 DPI group.

The mice in the control group did not exhibit any distinct pathological alterations and signs during the overall course of the experiment. However, *Lewis* lung carcinoma mice displayed time-dependent growth following the development of tumours. The sarcoma weights and volumes of the control, 7 DPI and 14 DPI group are shown in figure 1.

### 3.2. Optimization of the conditions of UPLC and QTOF/MS

In order to acquire better UPLC separation and peak shape of endogenous metabolites in mouse plasma, different mobile phase compositions and stationary phases were initially screened. It was found that the ACQUITY UPLC BEH C_18_ (100 × 2.1 mm, 1.7 µm) column proved better resolution and stronger retention ability for peaks of metabolite. To improve the resolution and enhance the peak shapes, 0.1% formic acid was selected in UPLC separation. Aiming to obtain better sensitivity for the endogenous metabolites in QTOF/MS spectra, the parameters of electrospray ionization including capillary voltage, source temperature, desolvation temperature and flow rate were tested. The parameters of QTOF/MS were optimized as follows: the temperature and flow rate of desolvation was 350°C and 600 l h^−1^, respectively; capillary voltage was 3.0 kV and −2.5 kV in positive and negative ion mode, respectively; ion source temperature was 120°C. After optimization of QTOF/MS^E^ parameters, Function 1 (collision energy set as 6 V) was employed to acquire the total ion current (TIC) chromatograms and Function 2 (collision energy ramping from 15 to 40 V) was used to obtain the fragment ions for different subclasses of endogenous metabolites in male mouse plasma. The representative TIC chromatograms of endogenous metabolites in male mouse plasma acquired in positive and negative ion modes are displayed in electronic supplementary material, figure S1.

### 3.3. Technical reproducibility of the analytical approach

The reproducibility of data in metabolomics experiments is very important to guarantee more reliable results. To ensure the data quality of UPLC-QTOF/MS, the periodic injection of QCP samples throughout the entire analysis was adopted as a method to guarantee quality in metabolic profiling. QCP sample has a number of merits, including being closest to the matrices of the biological sample and offering a dataset to assess the technical reproducibility of the analytical method [27].

**Figure 2.**
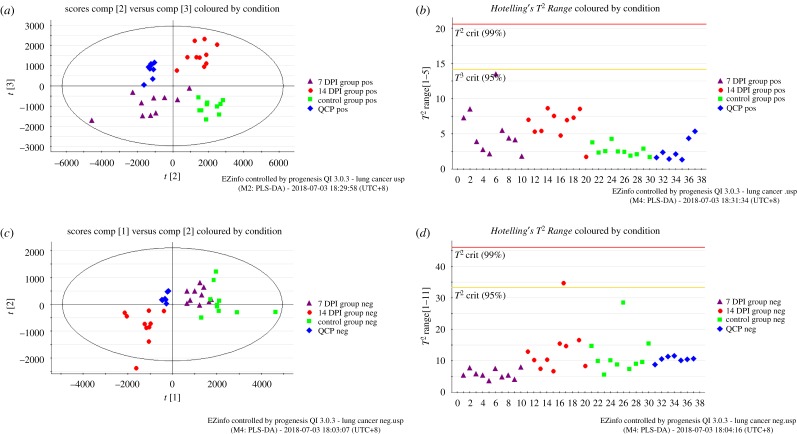
PCA score plot and *Hotelling's T^2^ Range* plot of all analysed samples in the 7 DPI model group, 14 DPI model group, control group and QCP samples. PCA score plot of all analysed samples in positive ion mode (*a*) with the statistical parameters (*R*^2^*X* = 94%, *Q*^2^ = 86%) and negative ion mode (*c*) with the statistical parameters (*R*^2^*X* = 97%, *Q*^2^ = 83%). The corresponding *Hotelling's T^2^ Range* plot in positive ion mode (*b*) and negative ion mode (*d*).

After data processing by Progenesis QI v. 3.0.3 software, 7193 and 5720 compound ions could be obtained in the positive and negative ion modes, respectively. More than 88.5% (6365 compound ions) and 83.9% (4800 compound ions) of variables were acquired by filtering peaks with CV less than 30% in the positive and negative ion modes, respectively. Then, the screened variables were subjected to EZinfo for multivariate data analysis. The score plots of PCA displayed the tightly clustered QCP samples and supplied pattern distinction for the 7 DPI model group, 14 DPI model group and control group in the positive (figure 2*a*) as well as negative ion modes (figure 2*c*), indicating their good analytical reproducibility throughout the experiment and confirming that variables observed in the sample were non-systematic but biologically relevant. In addition, the score plots of PCA showed that the 14 DPI model group exhibited a more obvious discrete tendency towards the normal mice from that of the 7 DPI model mice, showcasing a disease development linked to *Lewis* lung carcinoma. *Hotelling's T^2^ Range* plot was used to further check the quality of data. *Hotelling's T^2^ Range* plot (a value higher than the red line is a serious outlier) was employed to capture serious outliers in datasets and observe the effect of running order in positive as well as negative ion modes (figure 2*b*,*d*). Obviously, there was no serious outlier in all the samples. The score plots of PCA and *Hotelling's T^2^ Range* plots demonstrated that the repeatability and stability of the UPLC-QTOF/MS system were considered acceptable.

### 3.4. Metabolic alterations between model mice and controls at 7 DPI

**Figure 3.**
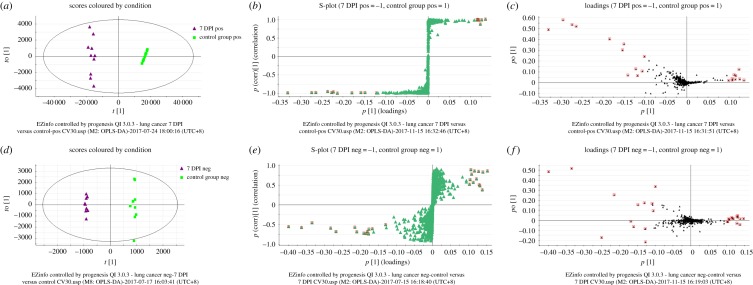
Score plot, corresponding S-plot and loading-plot from the OPLS-DA model between the 7 DPI group and control group. Score plot (*a*) generated from the OPLS-DA model between the 7 DPI and control group in positive ion mode (*R*^2^*Y* = 79%, *Q*^2^ = 58%), and corresponding S-plot (*b*) and loading-plot (*c*) from the OPLS-DA model. Score plot (*d*) generated from the OPLS-DA model between the 7 DPI and control group in negative ion mode (*R*^2^*Y* = 95%, *Q*^2^ = 87%), and corresponding S-plot (*e*) and loading-plot (*f*) from the OPLS-DA model.

**Figure 4.**
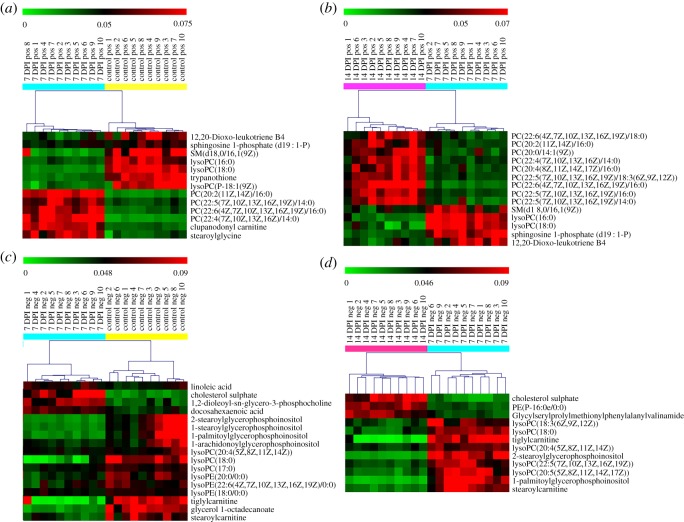
Heat maps of the significantly differential metabolites. Hierarchical clustering was used to separate individual samples (*X*-axis). *Y*-axis represents compounds. Metabolomic profile between different groups was shown a clear separation in the heat map. Normalized signal intensities are visualized as a colour spectrum in the heat maps. Red represents high expression, and green represents low expression of the dysregulated metabolites. Heat maps showcasing the significantly differential metabolites screened from the control group and 7 DPI group in positive (*a*) and negative (*c*) ion mode. Heat maps showcasing the significantly differential metabolites screened from the 14 DPI and 7 DPI group in positive (*b*) and negative (*d*) ion mode. Yellow colour represents control samples, cyan colour indicates 7 DPI samples and rose red colour indicates 14 DPI samples.

To better delineate the metabolic alterations in plasma between healthy mice and *Lewis* lung carcinoma mice, all observations obtained in the two ion modes were analysed by EZinfo. The model is considered reliable when *Q*^2^ is greater than 0.4. The score plots of OPLS-DA discriminated the 7 DPI group from the control groups in positive (figure 3*a*) and negative ion mode (figure 3*d*), respectively, which exhibited a satisfactory classification, implying that the differential metabolites contributed to good classification. The differential metabolites were visualized and filtered through the S-plots (figure 3*b*,*e*) and loading-plots (figure 3*c*,*f*) in both ion modes, respectively. In the S-plot and loading-plot, the further the distance of the differential metabolites from the original location, the higher the confidence level of the differential metabolites that contributed to the clustering observed in the score plots of OPLS-DA [28]. Then, the differential metabolites were selected from the S-plot and loading-plot in terms of the threshold of VIP values (VIP > 1.0) in OPLS-DA and *p*-values of *Welch's* *t*-test (*p* < 0.05). The heat maps displayed that the plasma metabolic states of *Lewis* lung carcinoma mice in the 7 DPI group deviated significantly from the corresponding controls in the positive (figure 4*a*) and negative ion mode (figure 4*c*), respectively. Thirteen and 17 differential metabolites were screened in positive and negative ion mode between the 7 DPI group and control group, respectively.

### 3.5. Metabolic variations in model mice between 7 DPI and 14 DPI

As the sarcoma grew, 14 and 12 differential metabolites were screened in positive and negative ion mode between 7 DPI and 14 DPI groups, respectively. The score plots of the OPLS-DA model discriminated the 14 DPI group from the 7 DPI group in positive (electronic supplementary material, figure S2A) and negative ion mode (electronic supplementary material, figure S2D), which showed satisfactory classification, implying that the differential metabolites contributed to the good separation. The differential metabolites were visualized and filtered by the S-plots (electronic supplementary material, figure S2B,E) and loading-plots (electronic supplementary material, figure S2C,F) in both ion modes, respectively. The heat maps also showed that the plasma metabolic states of 14 DPI group mice deviated significantly from 7 DPI in the positive (figure 4*b*) and negative ion mode (figure 4*d*), respectively.

**Figure 5.**
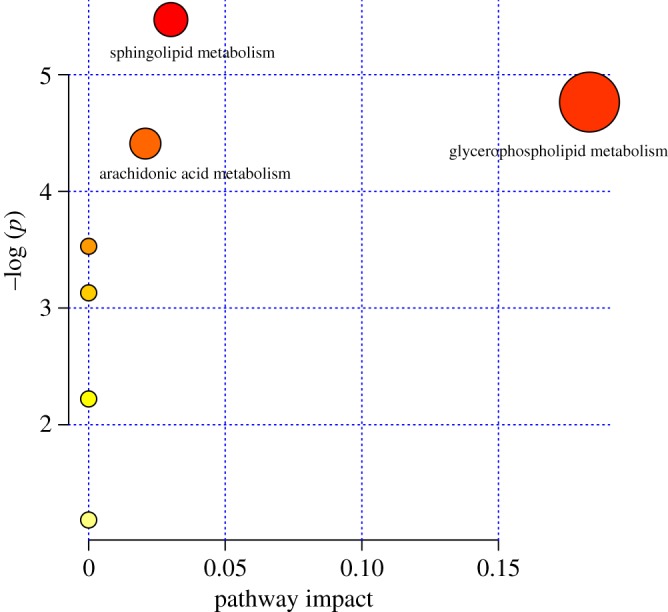
Pathway analysis of 15 dysregulated metabolites linked to the onset and development of *Lewis* lung carcinoma. The majority of these 15 dysregulated metabolites were involved in glycerophospholipid metabolism, sphingolipid metabolism and arachidonic acid metabolism.

As shown in table 1, 9 dysregulated metabolites under positive ion mode and 7 dysregulated metabolites under negative ion mode were detected at all the time points. Among them, lysoPC(18:0) was detected in both ion modes. The intensity differences of 15 potential biomarkers were evaluated by one way ANOVA. All pairwise multiple comparison procedures (Student–Newman–Keuls method) were performed with SPSS v. 18.0 (SPSS Inc., USA) to calculate the *p*-values among the control, 7 DPI and 14 DPI groups as shown in electronic supplementary material, figure S3. The related metabolic pathways of each dysregulated metabolite were also shown in the table through the KEGG online database (www.genome.jp/kegg/pathway.html). Most of these 15 dysregulated metabolites linked to the onset and development of *Lewis* lung carcinoma were involved in glycerophospholipid metabolism, sphingolipid metabolism, fatty acid metabolism and arachidonic acid metabolism. The pathway analysis module (integrating enrichment analysis) in the MetaboAnalyst v. 3.6 database was used to analyse the metabolic pathway of all 15 potential biomarkers, as shown in figure 5. The metabolic pathway (impact > 0.1) was considered to be the most significant pathway linked to the onset and development of *Lewis* lung carcinoma. As a result, glycerophospholipid metabolism was selected.

**Table 1.** Nine dysregulated metabolites under positive ion mode and 7 dysregulated metabolites under negative ion mode were detected throughout the onset and development of lung carcinoma. PC, phosphocholine; LysoPC, lysophosphatidylcholine; SM, sphingomyelin; FC, fold change.

features (*t*_R_–*m/z*)	adduct	formula	compound	*p*-value^a^	AUC^a^	log2(FC)^a^	metabolic pathway
*positive ion mode*							
11.39_496.3481	[M + H]^+^	C_24_H_50_NO_7_P	lysoPC(16:0)	2.26 × 10^−6^	0.99	−0.91	glycerophospholipid metabolism
13.95_524.3726	[M + H]^+^	C_26_H_54_NO_7_P	lysoPC(18:0)	5.40 × 10^−6^	0.94	−0.83	glycerophospholipid metabolism
23.58_703.5757	[M + H]^+^	C_39_H_79_N_2_O_6_P	SM(d18:0/16:1(9Z))	7.03 × 10^−4^	0.90	−0.53	sphingolipid metabolism
24.76_786.6020	[M + H]^+^	C_44_H_84_NO_8_P	PC(20:2(11Z,14Z)/16:0)	8.60 × 10^−5^	0.90	0.46	glycerophospholipid metabolism
22.46_760.5924	[M + 2Na + H]^+^	C_46_H_80_NO_8_P	PC(22:6(4Z,7Z,10Z,13Z,16Z,19Z)/16:0)	2.66 × 10^−4^	0.84	1.13	glycerophospholipid metabolism
23.98_782.5676	[M + H]^+^	C_44_H_80_NO_8_P	PC(22:4(7Z,10Z,13Z,16Z)/14:0)	4.79 × 10^−5^	0.96	1.03	glycerophospholipid metabolism
22.77_760.4027	[2M + ACN + Na]^+^	C_20_H_28_O_5_	12,20-dioxo-leukotriene B4	6.16 × 10^−3^	0.84	−0.29	arachidonic acid metabolism
27.18_832.5924	[2M + ACN + H]^+^	C_19_H_42_NO_5_P	sphingosine 1-phosphate (d19:1-P)	2.02 × 10^−2^	0.80	−0.76	sphingolipid metabolism
25.63_780.5592	[M + H]^+^	C_44_H_78_NO_8_P	PC(22:5(7Z,10Z,13Z,16Z,19Z)/14:0)	7.32 × 10^−6^	0.99	1.95	glycerophospholipid metabolism
*negative ion mode*							
24.52_465.3056	[M − H]^−^	C_27_H_46_O_4_S	cholesterol sulphate	5.46 × 10^−6^	1.00	1.58	fatty acid metabolism
14.46_485.2835	[2M − H]^−^	C_12_H_21_NO_4_	tiglylcarnitine	6.57 × 10^−6^	0.93	−0.49	fatty acid metabolism
10.46_1131.6713	[2M + FA − H]^−^	C_28_H_50_NO_7_P	lysoPC(20:4(5Z,8Z,11Z,14Z))	0.035437	0.78	−0.43	glycerophospholipid metabolism
13.91_568.3634	[M + FA − H]^−^	C_26_H_54_NO_7_P	lysoPC(18:0)	1.25 × 10^−6^	1.00	−2.33	glycerophospholipid metabolism
12.97_599.3215	[M − H]^−^	C_27_H_53_O_12_P	2-stearoylglycerophosphoinositol	6.08 × 10^−4^	1.00	−1.55	fatty acid metabolism
14.60_464.3167	[M + K − 2H]^−^	C_25_H_49_NO_4_	stearoylcarnitine	2.14 × 10^−4^	0.95	−0.80	fatty acid metabolism
10.79_571.2867	[M − H]^−^	C_25_H_49_O_12_P	1-palmitoylglycerophosphoinositol	6.26 × 10^−4^	1.00	−1.47	fatty acid metabolism

^a^Indicates the comparisons between 7 DPI and control group.

### 3.6. Identification of potential biomarkers

Receiver operating characteristic (ROC) curve analysis produced in the MetaboAnalyst v. 3.6 was used to confirm these potential biomarkers that significantly contributed to the discrimination of *Lewis* lung carcinoma mice from controls. Firstly, classical univariate ROC curve analysis were employed for the quantification of the predictive value of every endogenous biomarker. Using the area under the curve (AUC) of ROC, the values of specificity and sensitivity were calculated for each endogenous biomarker. The analysis confirmed nine significantly endogenous biomarkers in positive ion mode and seven significantly endogenous biomarkers in negative ion mode with AUC greater than 0.78 between the 7 DPI and control group, the data is shown in table 1.

**Figure 6.**
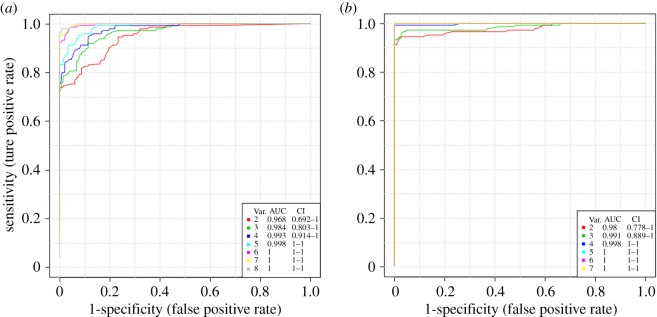
Comparison of dysregulated metabolites based on ROC analysis in positive ion mode and negative ion mode. The ROC curves were produced by Monte-Carlo cross validation based on balanced sub-sampling. Multivariate algorithm—support vector machines (SVM) was selected as classification and feature sorting method. (*a*) Biomarkers detected in positive ion mode. (*b*) Biomarkers detected in negative ion mode. Var. (variables) represents the number of selected features.

Then, multivariate exploratory ROC analysis was employed to evaluate the effectiveness of the combination of biomarkers for the diagnosis of *Lewis* lung carcinoma. The classification method and feature ranking method were support vector machines (SVM) and SVM built-ins, respectively. As shown in figure 6, the values produced in the AUC analysis were 1 (95% CI 1–1) for all nine biomarkers in positive ion mode and seven biomarkers in negative ion mode, respectively. The data illustrated that the identification of endogenous biomarkers, solely or in combination, can distinguish *Lewis* lung carcinoma from the corresponding controls with high accuracy and may be reliable indexes for *Lewis* lung carcinoma monitoring.

### 3.7. Biological information of biomarkers for *Lewis* lung carcinoma

Efforts to identify the biological markers associated with *Lewis* lung carcinoma not only help us to understand the pathologic mechanisms of the disease but also facilitate the effective screening of patients at earlier stages. In this context, our study was the first to describe and compare the specific plasma metabolic status of *Lewis* lung carcinoma mice at different stages with those of healthy mice based on the analysis of a significant number of biological samples.

Phosphoglycerolipid metabolites play important roles in the pathogenesis of tumours [29]. According to previous studies, the phosphoglycerolipid metabolites, characterized by the alteration of PCs, is a crucial aspect of tumour metabolism [30]. In addition to generating more matrices for the proliferation of cells, the glycerine phosphodiesterase-mediated metabolism of glycerophospholipids could also regulate the cellular signalling pathway via downstream products and cell migration through protein kinase C signalling [31]. In this study, the specific metabolites changed by *Lewis* lung carcinoma were mostly involved in glycerophospholipid metabolism (increased levels of PC(20:2(11Z,14Z)/16:0), PC(22:4(7Z,10Z,13Z,16Z)/14:0), PC(22:6(4Z,7Z,10Z,13Z,16Z,19Z)/16:0) and PC(22:5(7Z,10Z,13Z,16Z,19Z)/14:0); decreased levels of lysoPC(16:0), lysoPC(18:0) and lysoPC(20:4(5Z,8Z,11Z,14Z))). The increase of PCs implied that the onset and development of *Lewis* lung carcinoma led to alterations in cell proliferation and migration. Lecithin-cholesterol acyltransferase (LCAT) released into the blood by the liver synthesis exists in the free form or combined with lipoprotein and plays a catalytic enzyme role in the plasma [32]. LCAT can transform PCs into lysoPCs and cholesterol ester. Lysophospholipase D can transform lysoPCs into lysophosphatidic acids [33]. The decrease of lysoPCs may be because the tumour enhanced the activity of lysophospholipase D and inhibited the activity of LCAT.

Sphingolipids are important components of membrane lipids that take part in many cellular signalling pathways [34]. Sphingosine 1-phosphate(d19:1-P) and SM(d18:0/16:1(9Z)) are mainly converted from sphingosine via sphingosine kinase and could inhibit tissue oxidative injury. The reduction of the levels of sphingosine 1-phosphate(d19:1-P) and SM(d18:0/16:1(9Z)) indicated that the onset and development of *Lewis* lung carcinoma inhibited the activity of sphingosine kinase and aggravated the tissue oxidative damage.

12,20-Dioxo-leukotriene B4 was the metabolite of arachidonic acid released from PCs and phosphatidylethanolamine by phospholipase A2 (PLA2), released from phosphatidylinositol via phospholipase C (PLC), or released from diacylglycerol (DAG) under the effect of DAG lipase [35]. The decrease of 12,20-Dioxo-leukotriene B4 in *Lewis* lung carcinoma model mice indicated that the tumour may inhibit the activity of PLC, PLA2 or DAG lipase.

1-Palmitoylglycerophosphoinositol and 2-stearoylglycerophosphoinositol belong to chemical entities known as acyl-sn-glycerol-3-phosphoinositols. These are glycophosphoinositols where the glycerol is acylated only at position O-1 or O-2 with a fatty acid, respectively [36]. Tiglylcarnitine and stearoylcarnitine belong to a class of chemical entities called acyl carnitines. Acyl carnitines are organic compounds including a chain of a fatty acid with the carboxylic acid connected to carnitine via an ester bond [37]. The four endogenous components are involved in fatty acid metabolism and participate in lipid peroxidation. The decrease of 1-palmitoylglycerophosphoinositol, 2-stearoylglycerophosphoinosito, tiglylcarnitine and stearoylcarnitine in *Lewis* lung carcinoma model mice indicated that the tumour may dysregulate fatty acid metabolism and promote lipid peroxidation.

Cholesterol sulphate is a sterol sulphate in mammalian plasma. It is an integral part of the cell membrane with stable functions in the membrane, supporting platelet adhesion and involving signal transduction [38,39]. This compound belongs to the class of chemicals called cholesterols and derivatives. A previous metabolomic study showed that cholesterol sulphate was increased in lung cancer cells [40]. This metabolite can also change the activity of serine proteinases of the coagulation cascade [41]. Therefore, cholesterol sulphate may promote the interaction of platelets and circulating cells, including the spread of tumour cells in the blood. In addition, cholesterol sulphate can improve the protein hydrolysis activity of matrix metalloproteinase-7, which is related to the differentiation and proliferation of tumour cells.

## 4. Conclusion

In this study, a reliable untargeted metabolomic method based on UPLC-QTOF/MS in MS^E^ mode was proposed and applied to delineate metabolic changes in the male mouse plasma during lung carcinoma development. Significant metabolic alterations in the plasma levels of 9 and 7 potential biomarkers were observed in positive and negative ion mode, which were associated with the onset and development of lung carcinoma and involved in glycerophospholipid metabolism, sphingolipid metabolism, fatty acid metabolism and arachidonic acid metabolism. Collectively, the present study delineates distinct metabolic perturbations linked to the onset and development of lung carcinoma which may elucidate possible biological mechanisms of the disease.

## Supplementary Material

Electronic Supplemetary Information

## Data accessibility

Our data are available in an external publicly shared folder in Dryad Digital Repository: https://dx.doi.org/10.5061/dryad.1jq1313 [42].
