# Supplementary material for: Untargeted metabolomics profiles delineate metabolic alterations in mouse plasma during lung carcinoma development using UPLC-QTOF/MS in MSE mode
Source: R Soc Open Sci. 2018 Sep 19;5(9):181143. doi: 10.1098/rsos.181143 (PMC6170569; doi:10.1098/rsos.181143)
Supplement: Electronic Supplemetary Information [file rsos181143supp1.docx]

**Supplementary materials**

Untargeted metabolomics profiles delineate metabolic alterations in mouse plasma during lung carcinoma progression using UPLC-QTOF/MS in MS^E^ mode

Huan Wu^1,3 #^, Yang Chen^1,2,4 #^, Zegeng Li^1,2,3*^ and Xianhua Liu^1^

**Affiliation**

1 Key Laboratory of Xin'an Medicine, Ministry of Education, Anhui Province Key Laboratory of R&D of Chinese Medicine, Anhui University of Chinese Medicine, Hefei 230038, China. E-mail addresses: [li6609@126.com](mailto:li6609@126.com); Tel.: +86 551 65169269.

2 National Key Disciplines of Lung Disease of Anhui University of Chinese Medicine, State Administration of Traditional Chinese Medicine, Hefei 230038, China.

3 Institute of Pharmaceutics, Anhui Academy of Chinese Medicine, Hefei 230012, China.

4 Department of Traditional Chinese Medicine, The First Affiliated Hospital of Anhui Medical University, Hefei 230022, China.

# These authors contributed equally to this work; * The corresponding author.

**Correspondence**

* Prof. Zegeng Li. Postal address: Anhui University of Chinese Medicine, Meishan Road No. 103, Hefei 230038, China. Tel.: +86 551 65169269. E-mail addresses: li6609@126.com (Z. Li)

Supporting information

*^
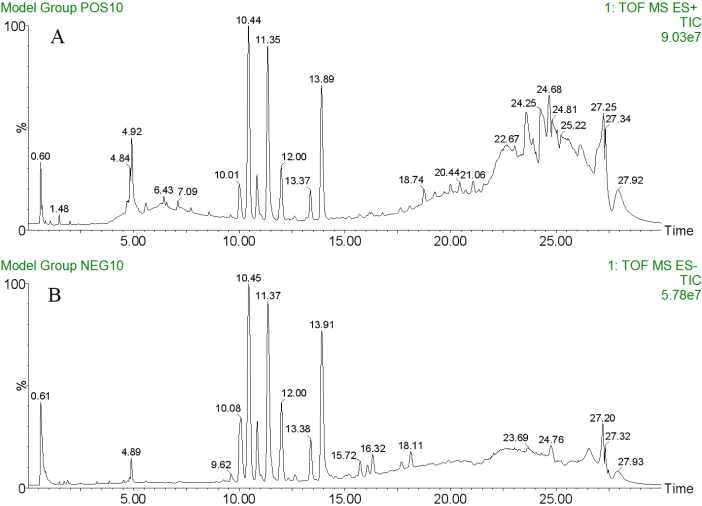
^*

**Figure S1. Representative UPLC-QTOF/MS total ion chromatograms (TIC) of Lewis lung carcinoma mouse plasma acquired in positive (A) and negative (B) ion mode**. X-axis represents the time (minute). Y-axis represents the relative intensity.


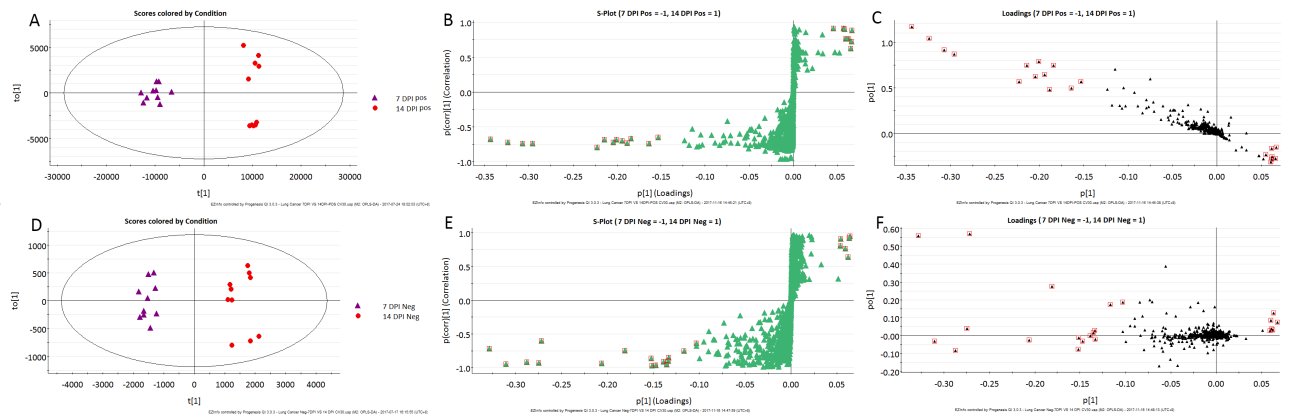


**Figure S2. Scores plot, and corresponding S-plot and lording-plot from OPLS-DA model between 14 DPI group and 7 DPI group.** Scores plots **(A)** generated from OPLS-DA model between 14 DPI and 7 DPI group in positive ion mode (*R*^2^*Y* = 86%, *Q*^2^ = 61%), and corresponding S-plot **(B)** and lording-plot **(C)** from OPLS-DA model. Scores plots **(D)** generated from OPLS-DA model between 14 DPI and 7 DPI group in negative ion mode (*R*^2^*Y* = 96%, *Q*^2^ = 82%), and corresponding S-plot **(E)** and lording-plot **(F)** from OPLS-DA model.


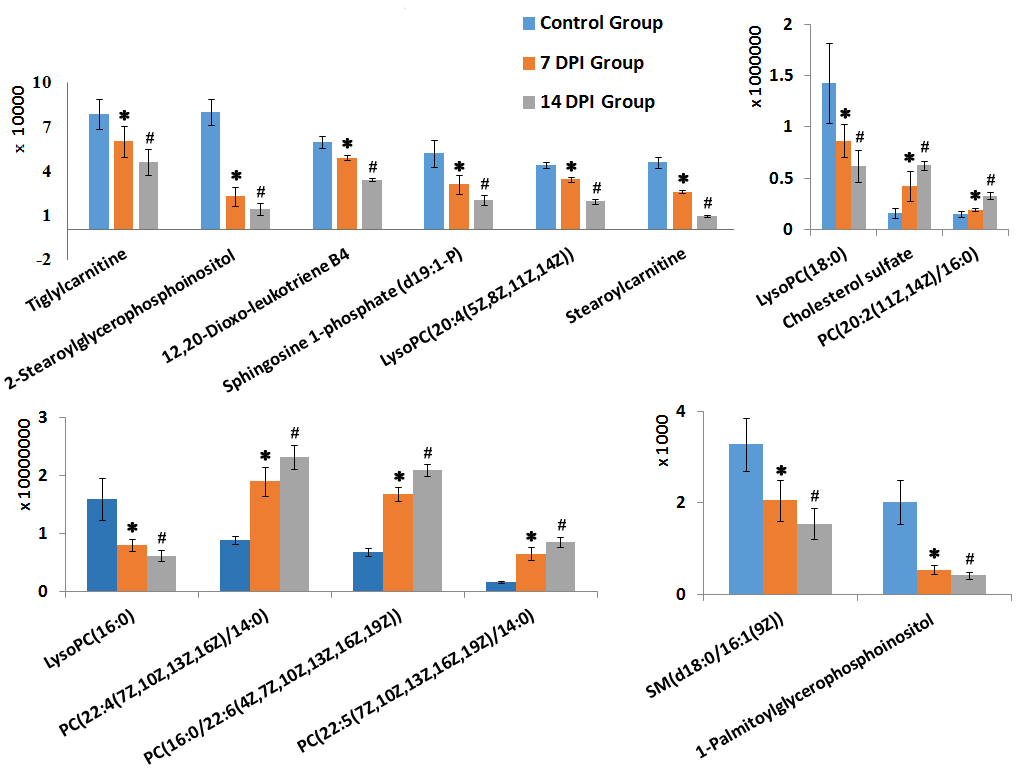


**Figure S3. Changes in the intensities of 15 potential biomarkers in the plasma sample from control to 7 day to 14 day.** **p* < 0.05: comparison between the control group and 7 DPI group; #*p* < 0.05: comparison between the 7 DPI group and 14 DPI group (n = 10 for each group)
